# Amoebicidal, anti-adhesive, and low-cytotoxic effects of *Mangifera indica* L. leaf extract against ocular *Acanthamoeba* spp.: First evidence supporting plant-based therapeutic potential

**DOI:** 10.14202/vetworld.2025.3322-3334

**Published:** 2025-11-06

**Authors:** Diana Mendonça, Hazel A. Tabo, Siriphorn Chimplee, Sónia M. R. Oliveira, Pattamaporn Kwankaew, Ana Paula Girol, Julieta Z. Dungca, Mazdida Sulaiman, Subha Bhassu, Muhammad Nawaz, Polrat Wilairatana, Christophe Wiart, Karma G. Dolma, Sunil Kayesth, Veeranoot Nissapatorn, Maria De Lourdes Pereira

**Affiliations:** 1 CICECO-Aveiro Institute of Materials, University of Aveiro, 3810-193 Aveiro, Portugal; 2 Department of Biology, University of Aveiro, 3810-193 Aveiro, Portugal; 3 Department of Biological Sciences, College of Science, De La Salle University-Dasmariñas, Cavite 4114, Philippines; 4 Department of General Education, School of Languages and General Education, Walailak University, Thai Buri, Nakhon Si Thammarat, 80160, Thailand; 5 Faculty of Dental Medicine, Catholic University, 3504-505 Viseu, Portugal; 6 Department of Medical Technology, School of Allied Health Sciences, Walailak University, Thai Buri, Nakhon Si Thammarat, 80160, Thailand; 7 Post Graduate Program in Structural and Functional Biology, Escola Paulista de Medicina (UNIFESP-EPM), Federal University of São Paulo, 04023-062, SP, Brazil; 8 Department of Biology, Institute of Biosciences, Humanities and Exact Sciences (Ibilce), São Paulo State University (UNESP), São José do Rio Preto, 15.054-999, SP, Brazil; 9 Experimental and Clinical Research Center (CEPEC), Padre Albino University Center (UNIFIPA), Catanduva, 15809-144, SP, Brazil; 10 School of Science and Technology, Centro Escolar University, Manila, 1005, Philippines; 11 Department of Chemistry, Faculty of Science, University of Malaya, Kuala Lumpur, 50603, Malaysia; 12 Institute of Biological Sciences, Faculty of Science, University of Malaya, Kuala Lumpur, 50603, Malaysia; 13 Department of Nano-Medicine Research, Institute for Research and Medical Consultations, Imam Abdulrahman Bin Faisal University, Dammam, 31451, Saudi Arabia; 14 Department of Clinical Tropical Medicine, Faculty of Tropical Medicine, Mahidol University, Bangkok, 10400, Thailand; 15 Department of Microbiology, Institute for Tropical Biology and Conservation, University Malaysia Sabah, Kota Kinabalu 88400, Malaysia; 16 Sikkim Manipal Institute of Medical Sciences, Sikkim Manipal University, Sikkim, India; 17 Department of Zoology, Medicinal Plant and Health Research Lab, Deshbandhu College, University of Delhi, Delhi 110019, India; 18 Futuristic Science Research Center-School of Science, World Union for Herbal Drug Discovery (WUHeDD), and Research Excellence Center for Innovation and Health Products (RECIHP), Walailak University, Nakhon Si Thammarat, 80160, Thailand; 19 Department of Medical Sciences, University of Aveiro, 3810-193 Aveiro, Portugal

**Keywords:** *Acanthamoeba*, amoebicidal activity, anti-adhesion, cytotoxicity, *Mangifera indica* L, ocular infection

## Abstract

**Background and Aim:**

*Acanthamoeba* spp. is free-living protozoa capable of causing severe infections, notably *Acanthamoeba* keratitis, which is difficult to manage due to cyst resistance and the cytotoxicity of current treatments. Plant-derived compounds represent a promising alternative strategy. This study investigated the amoebicidal, anti-adhesive, and cytotoxic properties of *Mangifera indica* L. (mango) leaf extract against ocularly relevant *Acanthamoeba* spp.

**Materials and Methods:**

Crude ethanolic leaf extract of *M. indica* was prepared and evaluated against *Acanthamoeba polyphaga* American Type Culture Collection (ATCC) 30461 and *Acanthamoeba castellanii* ATCC 50739. Minimum inhibitory concentration (MIC) and minimum parasiticidal concentration were determined for trophozoites and cysts. Morphological changes were analyzed by scanning electron microscopy (SEM). Anti-adhesion assays were conducted using polystyrene surfaces, with a commercial multipurpose contact lens (CL) solution as a control. Cytotoxicity was tested in Vero cells using the 3-(4,5-Dimethylthiazol-2-yl)-2,5-Diphenyltetrazolium Bromide assay to establish the minimum cytotoxic concentration.

**Results:**

The extract inhibited trophozoite growth at 2 mg/mL and demonstrated cysticidal activity at 4 mg/mL for *A. polyphaga* and 32 mg/mL for *A. castellanii*. SEM revealed disruption of trophozoite morphology, loss of acanthopodia, and surface perforations in cysts. At MIC levels, adhesion was reduced by >70%, and even at 1/8 MIC, inhibition remained above 50%, comparable to a commercial multipurpose solution. Cytotoxicity assessment showed >80% Vero cell viability at 0.125 mg/mL, indicating a favorable therapeutic window.

**Conclusion:**

This is the first report demonstrating amoebicidal and anti-adhesive effects of *M. indica* L. leaf extract against ocular *Acanthamoeba* species. The dual trophozoiticidal and anti-adhesive actions, combined with low cytotoxicity, highlight its potential for development as a plant-based therapeutic agent, particularly in ocular formulations or CL disinfectants. Future work should focus on phytochemical isolation, mechanistic studies, and novel delivery systems to enhance efficacy and safety.

## INTRODUCTION

*Acanthamoeba* species are ubiquitous free-living protozoa present in diverse ecological niches [1]. Their ability to alternate between two distinct life stages, an active trophozoite and a dormant, highly resistant cyst, confers remarkable resilience in clinical environments. Trophozoites are the invasive form responsible for host colonization and tissue damage, whereas cysts can endure adverse environmental conditions and resist most disinfectants and therapeutic agents, often necessitating prolonged treatment regimens [2, 3].

In humans, *Acanthamoeba* acts as an opportunistic pathogen, causing a wide spectrum of infections. Among these, *Acanthamoeba* keratitis (AK) is a vision-threatening ocular disease that may occur even in healthy individuals, particularly contact lens (CL) users. The global annual incidence of AK is estimated at 23,561 cases, though this figure likely underrepresents the true burden due to frequent misdiagnosis [4]. Other manifestations, such as granulomatous amebic encephalitis, cutaneous lesions, and rhinosinusitis, are more commonly seen in immunocompromised individuals and may progress to severe or fatal outcomes if untreated [5]. Importantly, cases of keratitis linked to *Acanthamoeba* are increasingly reported in both wild and domestic animals, broadening its relevance as a One Health concern [6].

Current management of AK relies on topical antiseptics, particularly biguanides (e.g., chlorhexidine and polyhexamethylene biguanide) and diamidines (e.g., propamidine isethionate and hexamidine) [7]. While these drugs are effective against trophozoites, their poor efficacy against cysts often results in persistent infection [8]. Moreover, the absence of specific anti-*Acanthamoeba* drugs necessitates aggressive and prolonged regimens, frequently associated with side effects such as ocular irritation, photophobia, excessive tearing, and reduced visual acuity [9]. Thus, there is a pressing need for alternative therapies capable of targeting both life stages with improved safety profiles.

*Mangifera indica* L. (mango), a tropical tree cultivated globally, has a long history of use in traditional medicine for treating various ailments [10]. Extracts from its leaves, bark, and fruits exhibit diverse pharmacological activities, including antimicrobial, anti-inflammatory, and antiparasitic effects, attributed to compounds such as mangiferin, gallic acid, catechins, and quercetin [11]. Notably, *M. indica* has demonstrated efficacy against protozoan pathogens such as *Leishmania* [12] and *Plasmodium* [13], suggesting its potential as a therapeutic candidate for amebic infections.

Despite decades of research, the management of *Acanthamoeba* infections, particularly AK, remains highly challenging. Existing therapeutic regimens rely on biguanides and diamidines, which demonstrate trophozoiticidal activity but have limited efficacy against the resilient cyst stage, often leading to recurrent or persistent infections. Moreover, prolonged administration of these agents is frequently associated with ocular toxicity and reduced patient compliance. Although several natural compounds and plant-derived extracts have been investigated for antimicrobial and antiparasitic properties, only a few studies have focused specifically on their effects against ocular *Acanthamoeba*. To date, no study has comprehensively evaluated the amoebicidal, cysticidal, and anti-adhesive properties of *M. indica* L. (mango) leaf extract against clinically relevant *Acanthamoeba* spp. The lack of such evidence represents a critical gap, particularly given the widespread availability, traditional medicinal use, and diverse bioactive phytochemistry of *M. indica*. Addressing this gap is essential to advance plant-based alternatives that could complement or replace current therapies while reducing adverse effects.

The present study aimed to provide the first experimental evidence of the amoebicidal, cysticidal, and anti-adhesive activities of *M. indica* L. leaf extract against pathogenic *Acanthamoeba polyphaga* and *Acanthamoeba castellanii*. Specifically, the work sought to: (i) determine the minimum inhibitory and parasiticidal concentrations of the extract for trophozoite and cyst forms; (ii) assess morphological alterations induced by treatment using scanning electron microscopy (SEM); (iii) evaluate the extract’s ability to inhibit adhesion of trophozoites to abiotic surfaces, mimicking an essential step in pathogenesis; and (iv) investigate cytotoxicity in mammalian cells to establish a therapeutic window. By integrating these approaches, the study not only addresses a major gap in anti-*Acanthamoeba* research but also explores the translational potential of *M. indica* as a natural, safe, and cost-effective candidate for ocular therapeutics and preventive formulations, such as CL disinfectant solutions.

## MATERIALS AND METHODS

### Ethical approval

All experimental procedures were performed in Biosafety Level 2 (BSL-2) standards and under the Committee of the Biosafety Guidelines for Scientific Research of Walailak University, Nakhon Si Thammarat, Thailand (Approval Ref. No.: WU-IBC-66-020). The study involved *in vitro* cultures of *A. polyphaga* (ATCC 30461) and *A. castellanii* (ATCC 50739) and cytotoxicity testing *in vero* cell lines; no vertebrate animals or human subjects were directly used.

### Study period and location

**Figure 1:**
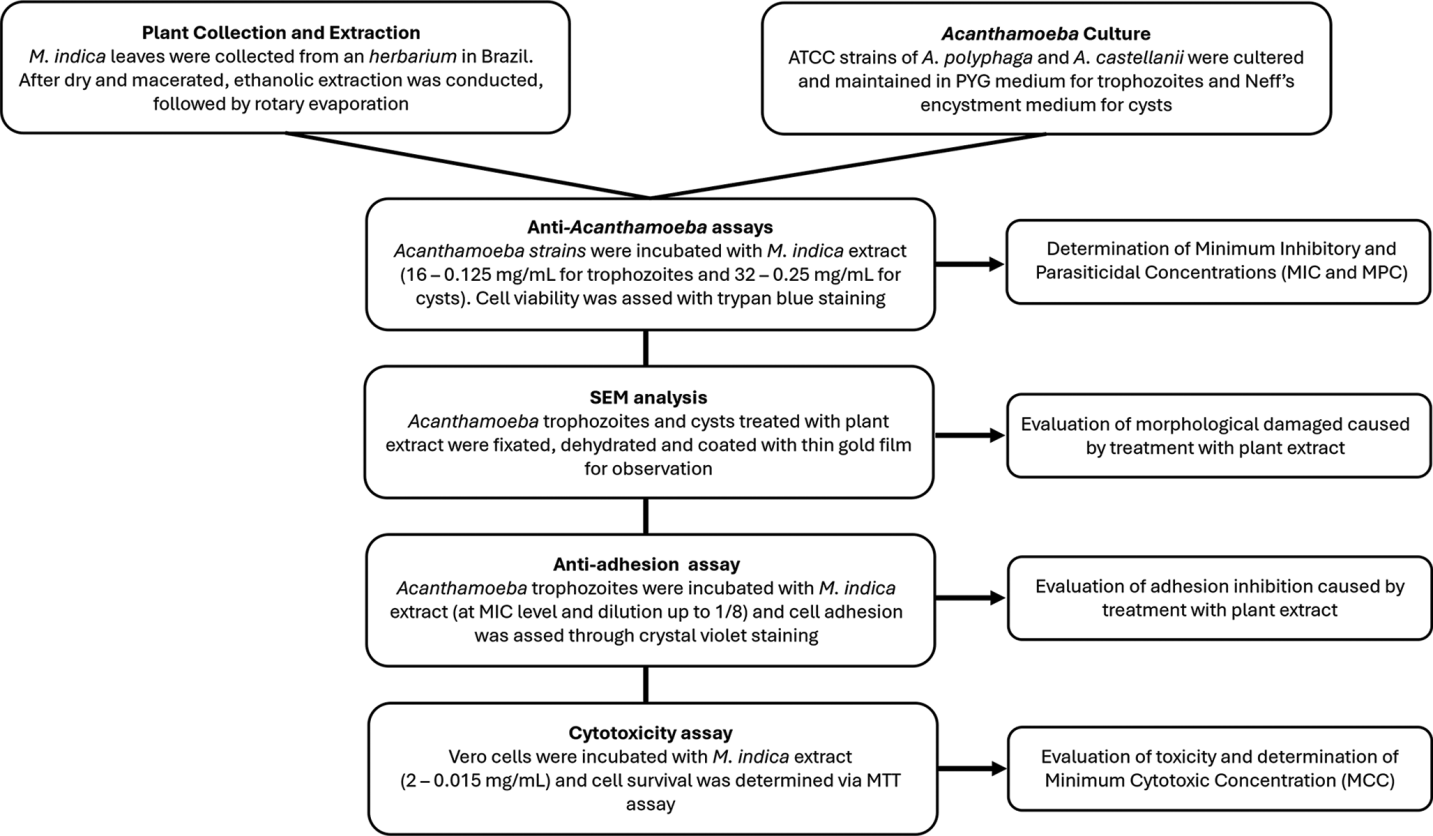
Experimental flowchart for evaluating anti-*Acanthamoeba* properties and cytotoxicity of *Mangifera indica*.

This study (Figure 1) was conducted from November to December 2024 at the Tropical Medicine Laboratory, Research Institute for Health Sciences, Walailak University, Thailand.

### Extraction of the plant material

*M. indica* L. samples were sourced from the Irina Delanova Gemtchujnicov Herbarium in Brazil (Table 1). The extraction process followed a previously established method with slight modifications [14]. The leaves were dried and finely ground into powder. A total of 50 g of the powdered plant was immersed in 200 mL of 95% ethanol (1:4 g/v) for 3 weeks, with agitation twice a week. The plant residue was removed using two layers of sterile gauze, and the resulting supernatant was filtered through filter paper (GE Healthcare Life Sciences, IL, USA). The ethanolic fractions were evaporated using a rotary evaporator (Buchi, New Castle, US) to obtain the crude extract. The extract was then maintained at 60°C for 3 days before being left at room temperature (RT; 26^o^C) to dry completely. Once dry, it was stored at 4°C and re-suspended in 100% dimethyl sulfoxide (DMSO) at a concentration of 200 mg/mL before use.

**Table 1:** Details of plant collection.

Registration	Collector	Day	Month	Year	Family	Genus	Species	Portuguese name
34712	Ferreira, M.I	30	8	2019	Anacardiaceae	*Mangifera*	*Indica*	Manga

### Acanthamoeba strains and cultivation

Two pathogenic *Acanthamoeba* strains were used in this study: *A. polyphaga* American Type Culture Collection (ATCC) 30461, obtained from the ATCC (ATCC, USA), and *A. castellanii* ATCC 50739, kindly provided by Dr. Rachasak Boonhok, Assistant Professor at the School of Allied Health Sciences, Walailak University. The culturing procedure was based on previously established methods [15, 16].

Both strains were propagated in T-25 cell culture flasks containing 7 mL of peptone-yeast-glucose (PYG) medium. The medium consisted of proteose peptone (20 g), glucose (18 g), and yeast extract (2 g) (all from HiMedia, Mumbai, India); sodium citrate dihydrate (1 g) (Sigma Chemical Co., St. Louis, MO, USA); MgSO_4_·7H_2_O (0.98 g), Na_2_HPO_4_·7H_2_O (0.355 g), and KH_2_PO_4_ (0.34 g) (all from Labscan, Bangkok, Thailand); and Fe(NH_4_)_2_(SO_4_)_2_·6H_2_O (0.02 g) (QRëC, New Zealand), all dissolved in 1,000 mL of distilled water. The pH was adjusted to 6.5. Cultures were maintained at RT in a dark environment for 72 h to facilitate the growth of mature trophozoites.

For cyst induction, trophozoites were harvested by centrifugation 1,968 × *g* for 5 min. The pellet was then washed twice with Neff’s encystment solution, consisting of KCl (7.455 g) (Daejung, Gyeonggi-do, Korea), Ammediol (2-amino-2-methyl-1,3-propanediol) (2.44 g) (Sigma Chemical Co., St. Louis, MO, USA), NaHCO_3_ (0.084 g) (HiMedia), MgSO_4_·7H_2_O (1.968 g) (Labscan, Bangkok, Thailand), CaCl_2_·2H_2_ (0.0588 g) (Sigma Chemical Co.), and 1,000 mL of distilled water, with the pH adjusted to 8.5. The washed amebae were suspended in 7 mL of Neff’s medium and incubated in the dark for 7 days at RT to allow complete encystation.

### Anti-Acanthamoeba activity assays

#### Minimum inhibitory concentration (MIC)

MIC assays were performed using the microtiter broth dilution technique, adapted from previously described methods [17]. Two-fold serial dilutions of the plant extract were prepared in 96-well microplates.

For the trophozoite tests, the concentrations ranged from 0.125 to 16 mg/mL in PYG medium. For cysts, the dilutions ranged from 0.25 to 32 mg/mL in Neff’s encystment medium. *Acanthamoeba* cells were inoculated into each well at a final density of 2 × 10^5^ cells per well.

A 2% DMSO solution served as a negative control, while chlorhexidine, serially diluted to final concentrations between 0.002 and 0.256 mg/mL, was included as a positive control. The microplates were incubated in the dark for 24 h at RT.

After incubation, cell viability was determined by staining with 0.2% trypan blue, and the percentage of viable cells was calculated using Equation 1 [16]. Separate assays were conducted for trophozoites and cysts, allowing the determination of the minimum trophozoite inhibitory concentration (MTIC) and minimum cyst inhibitory concentration (MCIC). These values represent the lowest concentration of the extract capable of inhibiting trophozoite or cyst viability by more than 90%, respectively.


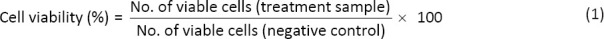


#### Minimum parasiticidal concentration (MPC)

MPC assay was performed following the same methodology described above for MIC assays, using the same concentrations of the plant extract and the same controls. The percentage of viable cells was determined daily over a 3-day period using trypan blue staining and calculated using Equation 1.

This assay required a prolonged incubation period, 3 consecutive days, and daily evaluation of cell viability. As in the MIC protocol, trophozoites and cysts were tested separately, enabling the determination of the MTC and MCC. These parameters represent the lowest extract concentrations that reduced viable trophozoite and cyst populations by more than 99.99% on the 3^rd^ day of treatment [14].

### SEM imaging

Treated *Acanthamoeba* trophozoites and cysts were collected from 96-well plates and transferred into sterile microcentrifuge tubes (Eppendorf, Hamburg, Germany). They were exposed to their respective MTIC and MCIC concentrations along with the positive and negative controls. The tubes were centrifuged at 1,073 × *g* for 5 min and the supernatant was removed. The resulting pellets were transferred to sterile, SEM-grade glass slides, which were then placed within a 24-well plate.

The samples were left to air dry at RT until they reached a semi-moist state, typically between 30 min and 1 h. Once partially dry, the samples were rinsed with 500 μL of phosphate-buffered saline (Oxoid Holdings, Hampshire, UK) and subsequently fixed in 500 μL of 2.5% glutaraldehyde for 12 h. Dehydration was performed using a graded ethanol series (20%–100%). The samples were dried after dehydration using a critical point dryer [16].

Finally, the specimens were mounted onto aluminum stubs and coated with a thin layer of gold in preparation for SEM analysis using a Zeiss SEM system (SEM-Zeiss, Munich, Germany).

### Anti-adhesion assay

The anti-adhesive activity of the plant extract was assessed using a modified protocol based on a previously described method [18], employing polystyrene 96-well microtiter plates as the substrate for cell attachment. The wells were supplemented with the plant extract at concentrations corresponding to MTIC and MCIC and dilutions up to 1/8, followed by inoculation with 4 × 10^5^ cells of each *Acanthamoeba* species.

A commercial multipurpose CL disinfectant solution (Multipurpose solution [MPS]; DUNA, Alcon Laboratories, TX, USA) served as the positive control, while 2% DMSO was used as the negative control. Plates were incubated for 24 h at RT in the dark.

After a 24-h incubation, non-adherent cells were removed by washing the wells with phosphate-buffered saline, after which the plates were air-dried at RT for 30 min. Adherent cells were stained with 0.1% crystal violet solution for 30 min. The excess stain was removed, and the wells were rinsed with distilled water and air-dried for an additional 30 min. Subsequently, 100 μL of 100% DMSO was added to each well.

Adhesion inhibition was quantitatively assessed by measuring the optical density at 570 nm using a microplate spectrophotometer. The inhibition percentages were calculated according to Equation 2.


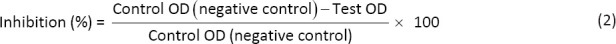


### Cytotoxicity assay and minimum cytotoxic concentration

The cytotoxicity of *M. indica* L. was evaluated using the 3-(4,5-dimethylthiazol-2-yl)-2,5-diphenyltetrazolium bromide (MTT) colorimetric assay [14]. Vero cells (ECACC 84113001, RRID: CVCL_0059; Salisbury, UK), provided by Associate Professor Dr. Chuchard Punsawad (School of Medicine, Walailak University), were cultured in Dulbecco’s Modified Eagle’s Medium (DMEM; Merck KGaA, Darmstadt, Germany) supplemented with 10% fetal bovine serum (FBS) (FBS; Sigma-Aldrich, St. Louis, MO, USA) and 1% penicillin-streptomycin solution (Thermo Fisher Scientific, MA, USA). Cultures were maintained at 37°C in a humidified atmosphere containing 5% CO_2_. After reaching approximately 90% confluence, cells were enzymatically detached using 2.5% trypsin-ethylenediaminetetraacetic acid (Thermo Fisher Scientific) and incubated for 5–10 min to ensure complete detachment.

Subsequently, cells were resuspended and seeded into 96-well plates at a density of 1.5 × 10^4^ cells/mL, followed by a 24-h incubation to facilitate cell attachment.

To evaluate cytotoxicity, a 2 mg/mL stock concentration of the plant extract was added to the first well and subjected to two-fold serial dilutions to yield concentrations ranging from 0.015 to 2 mg/mL, incorporating the previously obtained MIC/MPC levels. Wells treated with 1% DMSO served as negative controls. After a 24-h exposure period, the MTT assay was performed to evaluate the metabolic activity of *Acanthamoeba* cells. Specifically, 100 μL of 0.5 mg/mL MTT solution (Thermo Fisher Scientific) was added to each well and incubated for 1 h at 37°C. The MTT solution was removed and replaced with 100% DMSO.

The absorbance was measured at 570 and 650 nm using a microplate spectrophotometer. Cell viability was determined and expressed as a percentage relative to the negative control using Equation 3. The minimum cytotoxic concentration was defined as the lowest plant extract concentration that reduced cell viability by 20%.


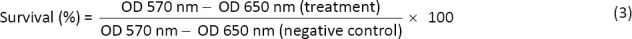


### Statistical analysis

All experimental assays were conducted in triplicate (i.e., measurements of the same sample were repeated). The data were compiled and processed using Microsoft Excel version 2506 (Microsoft Corporation, Redmond, Washington, USA). Results are presented as mean ± standard deviation. Statistical analysis across all experimental groups was performed using one-way analysis of variance followed by Tukey’s honestly significant difference test. p < 0.05 was considered statistically significant and was appropriately reported throughout the study.

## RESULTS

### MIC and MPC

The *M. indica* L. extract inhibited the growth of *Acanthamoeba* trophozoites at a concentration of only 2 mg/mL in *A. polyphaga* and *A. castellanii*. For cysts, the MIC ranged from 4 to 32 mg/mL for *A. polyphaga* and *A. castellanii*, respectively. *A. polyphaga* exhibited greater overall susceptibility, requiring a lower concentration to inhibit cyst growth.

However, *A. castellanii* cysts displayed more resistance to treatment, with significant inhibition only at the maximum concentration of 32 mg/mL. Chlorhexidine, used as a positive control, showed superior anti-*Acanthamoeba* efficacy, ranging from 0.004 to 0.008 mg/mL against both trophozoite and cyst forms (Table 2).

**Table 2:** Minimum inhibitory concentration and minimum parasiticidal concentration for *Acanthamoeba polyphaga* and *Acanthamoeba castellanii* cysts and trophozoites.

*Acanthamoeba* strain	*Mangifera indica* L.				CHX			
	
	MTIC	MCIC	MTCC	MCCC	MTIC	MCIC	MTCC	MCCC
*Acanthamoeba polyphaga* ATCC 30461	2	4	2	4	0.004	0.004	0.004	0.004
*Acanthamoeba castellanii* ATCC 50739	2	32	2	32	0.004	0.008	0.004	0.008

MTIC = Minimum trophozoite inhibitory concentration (mg/mL), MCIC = Minimum cyst inhibitory concentration (mg/mL), MTCC = Minimum trophocidal concentration (mg/mL), MCCC = Minimum cytocidal concentration (mg/mL), CHX = Chlorhexidine

**Figure 2:**
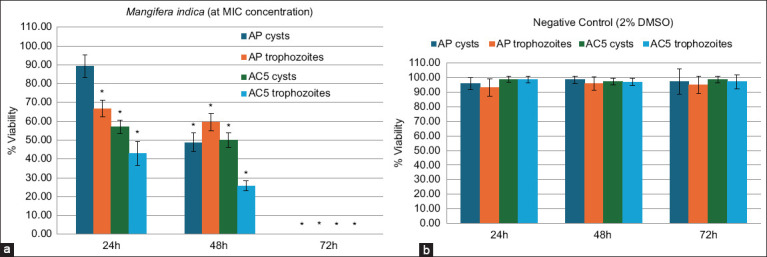
Viability percentage for the parasiticidal assay for cysts and trophozoites of *Acanthamoeba polyphaga* and *Acanthamoeba castellanii* treated with (a) plant extract corresponding to the determined minimum inhibitory concentration compared to the (b) negative control. Asterisks (*) indicate the groups that differ significantly (p < 0.05) from the control group, AP = *Acanthamoeba polyphaga*, AC = *Acanthamoeba castellanii*.

MPC concentrations were equal to MIC values (Table 2), indicating that these concentrations could eliminate viable growth after 3 days of exposure, resulting in 0% viability for trophozoites and cysts (Figure 2).

### SEM analysis

**Figure 3:**
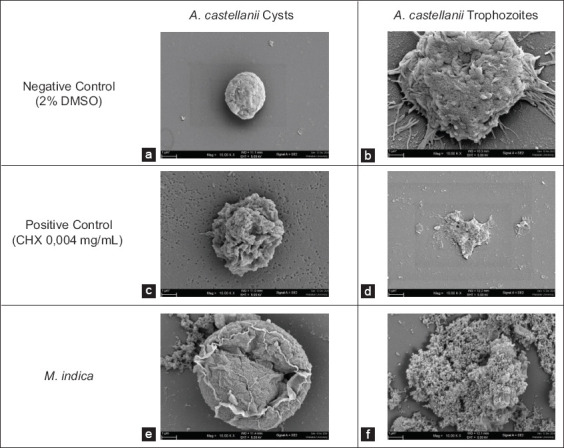
Scanning electron microscopy imaging of (a) Acanthamoeba castellanii cysts used as negative control, (b) trophozoites used as negative control, (c) cysts used as positive control, (d) trophozoites used as positive control, and (e) cysts treated with 32 mg/mL plant extract, and (f) trophozoites treated with 2 mg/mL plant extract (minimum trophozoite inhibitory concentration).

**Figure 4:**
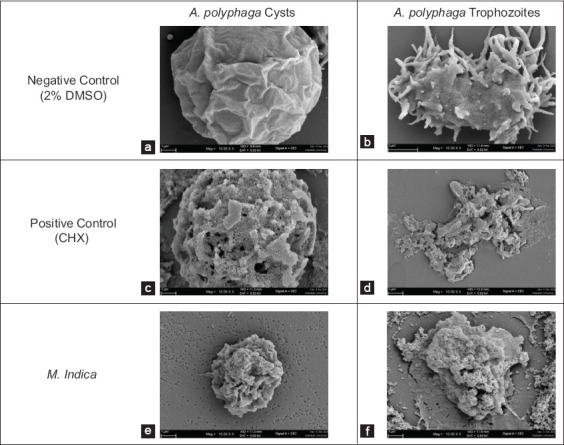
Scanning electron microscopy imaging of (a) *Acanthamoeba polyphaga* cysts used as negative control, (b) trophozoites used as negative control, (c) cysts used as positive control, (d) trophozoites used as positive control, (e) cysts treated with 4 mg/mL (minimum cyst inhibitory concentration) of plant extract, and (f) trophozoites treated with 2 mg/mL (minimum trophozoite inhibitory concentration) of plant extract.

The morphological changes in *Acanthamoeba* cysts and trophozoites induced by the MIC of the plant extract were evaluated using SEM. In the negative control (2% DMSO), the cells exhibited smooth surfaces without perforations, and the trophozoites retained intact acanthopodia (Figures 3a and b; 4a and b), indicating no structural damage.

A lower concentration (MCIC: 4 mg/mL) caused surface perforations and cyst shrinkage in *A. polyphaga* (Figure 4e). The trophozoites displayed transcellular perforations and the absence of acanthopodina, indicating severe damage and loss of viability (Figure 4f).

In contrast, chlorhexidine-treated cells (positive control) showed extensive perforations and morphological integrity loss in both cysts and trophozoites (Figures 3c and d; 4c and d).

Treatment with *M. indica* L. extract induced structural alterations. Only a disruption of the outer membrane was observed in *A. castellanii* cysts (MCIC: 32 mg/mL), suggesting higher resistance to the extract (Figure 3e). In trophozoites, 2 mg/mL MTIC led to complete acanthopodia and cell structure loss (Figure 3f).

### Anti-adhesion assay

**Figure 5:**
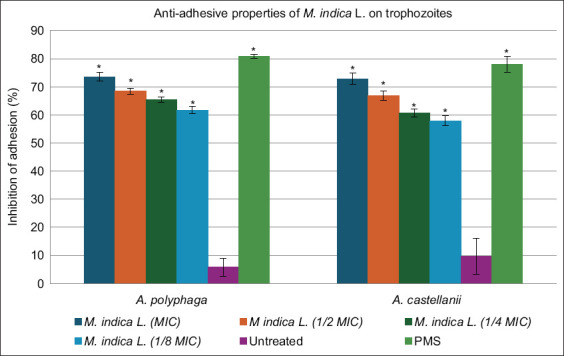
*Mangifera indica* L. extract dilutions compared with the positive control (multipurpose solution) and untreated cells with the standard deviation for each group. Asterisks (*) represent groups that statistically differ (p < 0.5) from the negative control (untreated).

Following the determination of the MIC and assessment of its impact on the morphology of *Acanthamoeba* cells, the anti-adhesive properties of the *M. indica* L. extract against trophozoites were evaluated. At MIC, the extract significantly inhibited the adhesion of *A. polyphaga* and *A. castellanii* trophozoites (Figure 5) by more than 70%.

A concentration-dependent decrease in adhesion inhibition was noted, although inhibition remained comparable to MPS even at 1/8 MIC, still allowing more than 50% adhesion inhibition for both species. Untreated *Acanthamoeba* cells showed high adhesion levels, whereas treatment with the extract or MPS significantly reduced adhesion (p < 0.05).

### Cytotoxicity assay and minimum cytotoxic concentration

**Figure 6:**
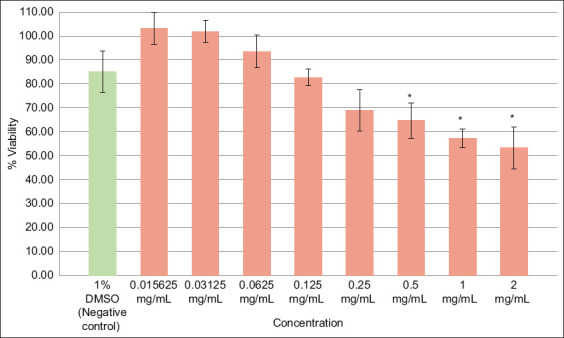
Cytotoxicity of *Mangifera indica* L. on Vero cells. The percentage of viability is represented for each concentration tested (extract in orange and negative control in green) (standard deviation is represented for each). Asterisks (*) represent the groups that differed significantly (p < 0.05) compared to the negative control.

At 0.125 mg/mL, the *M. indica* L. extract maintained more than 80% cell viability, which was established as the minimum cytotoxic concentration (Figure 6). Significant reductions in viability (p < 0.05) were observed only at the highest concentrations of 2, 1, and 0.5 mg/mL relative to the negative control.

## DISCUSSION

To the best of our knowledge, this is the first study to report the amoebicidal and anti-adhesive activities of *M. indica* L. leaf extract against ocular *Acanthamoeba* species. The crude extract exhibited amoebicidal activity against *A. polyphaga* and *A. castellanii* trophozoite and cyst forms.

### Amoebicidal activity and MIC/MPC findings

The MIC for trophozoites of both species was 2 mg/mL, whereas cysts required significantly higher concentrations, ranging from 4 mg/mL for *A. polyphaga* to 32 mg/mL for *A. castellanii*. These findings suggest a markedly higher resistance in *A. castellanii* cysts, a trend consistent with earlier studies that highlight the species’ robust encystment process and its protective double-layered cell wall, particularly rich in cellulose [19, 20]. Such morphology, combined with enhanced expression of drug efflux mechanisms such as ATP-binding cassette transporters [21], likely contributes to the reduced susceptibility of *A. castellanii* cysts to the plant extract.

Furthermore, the obtained MIC values also reveal promising results when compared to a study by Nayeri *et al.*, which reported amoebicidal activity only at 10 mg/mL of *Rumex obtusifolius* leaf extract and only 28.6% trophozoite killing and 0% cyst killing with seed extract [22].

### Morphological alterations observed by SEM

SEM provided further insight into the morphological alterations induced by the extract. In trophozoites of both species, the treatment led to the complete elimination of acanthopodina, indicating a loss of motility and adherence capacity.

In contrast, SEM images of *A. castellanii* cysts revealed damage confined to the outer membrane, suggesting that the inner wall remained largely intact, further emphasizing the resilience of this species. Moreover, SEM imaging allows us to hypothesize that the principle of action of *M. indica* L. relies on membrane disruption, as extensive damage is observed in both species.

### Anti-adhesion properties

In the anti-adhesion assays, *M. indica* L. extract significantly reduced the ability of *Acanthamoeba* trophozoites to adhere to surfaces compared with untreated controls, although its efficacy was lower than that of the positive control MPS. However, these findings are promising, as adhesion is a critical step in the pathogenesis of AK, particularly in CL-associated infections [23]. Thus, the inhibition of adhesion combined with the amoebicidal properties of this extract could contribute to preventative or therapeutic strategies.

### Cytotoxicity assessment

The extract also exhibited low cytotoxicity in the tested cell line. The minimum cytotoxic concentration was 0.125 mg/mL, indicating a relatively high threshold before the occurrence of adverse effects in host cells [24]. This level of cytocompatibility suggests a favorable therapeutic window for further exploration, especially in topical applications where direct exposure can be optimized to maximize anti-*Acanthamoeba* activity while minimizing toxicity [25].

### Therapeutic potential of *M. indica*

Collectively, these results demonstrate the potential of *M. indica* L. as a source of bioactive compounds for the management of *Acanthamoeba* infections. Moreover, the dual amoebicidal/anti-adhesive action, combined with the low toxicity, highlights that this extract could be applied as an alternative to toxic biguanides and diamidines in CL disinfectant solutions.

However, as this study used crude extracts, future efforts should focus on phytochemical analysis and compound isolation. Previous reports have identified various biologically active constituents in *M. indica* L., with its major constituents being mangiferin, gallic acid, catechins, and quercetin [26, 27], many of which are known for antimicrobial, anti-inflammatory, and antiparasitic properties [11]. Identifying the specific components responsible for anti-*Acanthamoeba* effects would allow for the targeted development of more potent and selective therapies.

### Role of bioactive compounds

Although no direct studies have been conducted against *Acanthamoeba* for the xanthone mangiferin, its known medicinal properties suggest potential efficacy. Moreover, α-mangostin, also a xanthone derived from *Garcinia mangostana* L., has shown amoebicidal effects against both trophozoites and cysts [28].

Gallic acid has demonstrated significant anti-*Acanthamoeba* activity; for instance, gallic acid encapsulated in PLGA nanoparticles showed up to 90% inhibition of trophozoites at 100 μg/mL, with reduced cytotoxicity compared to free gallic acid [29]. Catechins, particularly epigallocatechin gallate, also exhibited anti-*Acanthamoeba* activity [30].

Studies using green tea extracts of *Camellia sinensis* (L.) Kuntze, rich in catechins, reported significant inhibition of trophozoite replication and encystation, with low cytotoxicity to human corneal epithelial cells [31]. These compounds are now targets for further isolated studies against *Acanthamoeba* because they could be responsible for the results obtained in this study.

Moreover, molecular docking studies and molecular dynamic simulation analysis are being conducted to highlight possible interactions with *Acanthamoeba* essential proteins. These approaches will allow the understanding of the behavior of the mentioned phytochemicals to assess if they are eligible for further experimental work.

## CONCLUSION

This study provides the first evidence of the amoebicidal and anti-adhesive activities of *M. indica* L. leaf extract against ocular *Acanthamoeba* species. The crude extract exhibited potent inhibitory effects, with MIC values of 2 mg/mL for trophozoites and 4–32 mg/mL for cysts, highlighting the relatively higher resistance of *A. castellanii* cysts compared to *A. polyphaga*. SEM analysis confirmed that the extract induced significant morphological damage, including acanthopodia loss in trophozoites and membrane disruption in cysts. In addition, the extract significantly reduced trophozoite adhesion by more than 70% at MIC, a crucial step in the pathogenesis of AK. Importantly, cytotoxicity assays demonstrated a favorable safety profile, with more than 80% viability maintained at 0.125 mg/mL, suggesting a wide therapeutic window.

The practical implications of these findings are noteworthy. The dual amoebicidal and anti-adhesive properties of *M. indica* extract, coupled with low cytotoxicity, underscore its potential as a natural, safer alternative to conventional biguanides and diamidines commonly used in CL disinfectant solutions. Such an approach may provide a preventive or adjunctive therapy, particularly for CL-associated keratitis where resistance to standard treatments is a growing concern.

A major strength of this study lies in its multipronged evaluation, combining MIC/MPC assays, SEM imaging, anti-adhesion assays, and cytotoxicity profiling, which together provide a comprehensive insight into the therapeutic potential of *M. indica* L. However, some limitations should be acknowledged. The study was based on crude extracts without isolation of active compounds, and results were not corroborated with molecular docking or dynamic simulation analyses. Furthermore, *in vitro* assays cannot fully replicate the complexity of ocular environments, which may affect clinical translation.

Future research should focus on the phytochemical characterization of the extract to identify specific bioactive constituents such as mangiferin, gallic acid, catechins, and quercetin, which are likely contributors to the observed effects. Integration with nanotechnology-based delivery systems, such as nanoparticle encapsulation, should also be explored to enhance bioavailability, controlled release, and targeted delivery [32–34]. *In vivo* studies and ocular surface models will be crucial in validating efficacy and safety under conditions that closely mimic clinical applications.

*M. indica* L. leaf extract demonstrates promising anti-*Acanthamoeba* activity with low cytotoxicity, positioning it as a potential candidate for future development of plant-derived therapeutics and preventive strategies. While further validation is required, this study sets a strong foundation for advancing natural product-based interventions against drug-resistant and CL-related *Acanthamoeba* infections.

## AUTHORS’ CONTRIBUTIONS

DM, HAT, and SC: Performed the experiments. DM and SC: Performed the data analysis and drafted the manuscript. VN and MLP: Supervised the study SMRO, PK, APG, JZD, MS, SB, MN, PW, CW, KGD, SK, VN, and MLP: Validated and curated data and reviewed the manuscript. All authors have read and approved the final version of the manuscript.
